# Stimuli Responsive Nitric Oxide-Based Nanomedicine for Synergistic Therapy

**DOI:** 10.3390/pharmaceutics13111917

**Published:** 2021-11-12

**Authors:** Yijun Zhao, Xumei Ouyang, Yongjun Peng, Shaojun Peng

**Affiliations:** 1 Zhuhai Institute of Translational Medicine, Zhuhai Precision Medical Center, Zhuhai People’s Hospital (Zhuhai Hospital Affiliated with Jinan University), Zhuhai 519000, China; zhaoyijun94@163.com (Y.Z.); oyxumei@zju.edu.cn (X.O.); 2 The Department of Medical Imaging, Zhuhai People’s Hospital (Zhuhai Hospital Affiliated with Jinan University), Zhuhai 519000, China

**Keywords:** gas therapy, stimuli responsive, drug delivery, nanomedicine, nitric oxide

## Abstract

Gas therapy has received widespread attention from the medical community as an emerging and promising therapeutic approach to cancer treatment. Among all gas molecules, nitric oxide (NO) was the first one to be applied in the biomedical field for its intriguing properties and unique anti-tumor mechanisms which have become a research hotspot in recent years. Despite the great progress of NO in cancer therapy, the non-specific distribution of NO in vivo and its side effects on normal tissue at high concentrations have impaired its clinical application. Therefore, it is important to develop facile NO-based nanomedicines to achieve the on-demand release of NO in tumor tissue while avoiding the leakage of NO in normal tissue, which could enhance therapeutic efficacy and reduce side effects at the same time. In recent years, numerous studies have reported the design and development of NO-based nanomedicines which were triggered by exogenous stimulus (light, ultrasound, X-ray) or tumor endogenous signals (glutathione, weak acid, glucose). In this review, we summarized the design principles and release behaviors of NO-based nanomedicines upon various stimuli and their applications in synergistic cancer therapy. We also discuss the anti-tumor mechanisms of NO-based nanomedicines in vivo for enhanced cancer therapy. Moreover, we discuss the existing challenges and further perspectives in this field in the aim of furthering its development.

## 1. Introduction

Globally, cancer is one of the most serious diseases threatening human health. Despite the rapid advances made in diagnosis and treatment technologies, it remains a great challenge due to the heterogeneity and complexity of tumors themselves [1,2,3,4]. Therefore, basic and clinical studies for effective cancer treatment are still in desperate need in order to improve the health of human beings [5,6,7,8]. Presently, common treatments for tumors are chemotherapy, radiotherapy, surgery and biological treatment [9,10,11]. However, clinical outcomes have proven that these treatments exhibit certain limitations and side effects such as an off-targeting phenomenon and damage to normal cells, which hinder the significant improvement of the survival rate of patients [12,13,14,15]. On this accession, it is significant to explore new types of tumor treatment methods for superior therapeutic efficiency and reduced side effects. Compared with traditional anti-tumor methods, gas therapy has received widespread attention as an emerging and promising therapeutic approach to cancer treatment [5]. Many gas molecules such as nitric oxide (NO), carbon monoxide (CO), hydrogen sulfide (H_2_S) and hydrogen selenide (H_2_Se) have been proven to participate in the physiological and pathological processes of living subjects as signal transduction molecules [16]. Therefore, regulating the gas concentrations in the tumor microenvironment (TME) could profoundly affect the occurrence and development of tumors [17]. To begin with, previous studies have demonstrated that therapeutic gas molecules could effectively pass through the biological barriers owing to their diffusivity [18]. Secondly, some therapeutic gas molecules could reverse the Warburg effect in tumor tissue, thereby inhibiting the activity of tumor cells without harming the normal cells at certain concentrations [19,20]. In addition, the anti-tumor effects of gas therapy are multiple. For example, NO has been demonstrated to strengthen the enhanced permeability and retention (EPR) effect by vasodilation, which could enhance the accumulation efficiency of nanomedicines [21]. Moreover, NO, CO and sulfur dioxide (SO_2_) could enhance the effect of chemotherapy by overcoming the multidrug resistance (MDR) of tumor cells [22,23,24]. Furthermore, hydrogen (H_2_) has been found to kill tumor cells and protect normal cells at the same time at proper concentrations [25]. In addition, our research group also achieved an anti-tumor effect by using responsively released H_2_Se which effectively inhibited tumor metastasis by down-regulating the expression of metastasis-related proteins [26]. Finally, some gas molecules could enhance the effect of radiotherapy, photodynamic therapy (PDT) or immunotherapy by increasing the oxygenation ability of tumor cells and so on [27,28,29,30,31]. Therefore, as a new and promising anti-tumor method, gas therapy has increasingly received attention in anti-tumor therapy and presented itself as a new opportunity for the treatment of cancer [18,22,32].

To date, commonly used gas molecules in cancer therapy have been NO, CO, H_2_, H_2_S, SO_2_ and H_2_Se, among which NO was the first one to be developed in the biomedicine field [33,34,35]. In 1998, Dr. Louis J. Ignarro won the Nobel Prize for discovering the role of NO in the cardiovascular system. Since then, numerous studies have focused on the biomedical applications of NO [36,37]. It has been found that NO plays an important role in the cardiovascular system, respiratory system, nervous system, immune system, antibacterial treatment, wound healing and other biomedical fields [38,39,40]. It is especially in the field of cancer treatment that an increasing number of studies have demonstrated that NO presents direct or indirect anti-tumor effects at different concentrations [41,42,43,44]. The main anti-tumor mechanisms of NO are listed as follows: (1) NO firmly binds to the blood iron center of many proteins and thus inhibits the mitochondrial respiratory metabolism of cancer cells [5,33,45]; (2) NO combines with intracellular superoxide anions to produce free radicals that damage the DNA of tumor cells [33,39]; (3) NO could effectively dilate the blood vessels at the tumor site leading to an enhanced EPR effect [27,28,46]. The main mechanism is the combination of NO to the heme group of the soluble guanylyl cyclase (sGC) which enhances the activity of sGC, thereby enhancing the activity of protein kinase G and reducing the intracellular Ca^2+^ level, leading to a vasodilation effect [47]. Meanwhile, NO can also reduce platelet aggregation by decreasing Ca^2+^ level in platelets, thus inhibiting the integrity of tumor blood vessels and ultimately enhancing the EPR effect [48]; (4) P-glycoprotein (P-gp), one of the adenosine triphosphate (ATP) binding cassette transporters, is over-expressed in tumor cell membranes [30,49]. NO can nitrate tyrosine residues in P-gp and its transport function, thus inhibiting the drug outflow from tumor cells and overcoming the MDR of tumor cells to chemotherapy [48]; (5) NO could also be applied as a radiosensitizer to improve the efficacy of radiotherapy for hypoxic tumors [50,51]. Therefore, NO-based gas therapy has been widely applied in tumor therapy with multiple functions and applications.

Despite the many advantages of the therapeutic effect of NO gas in tumor therapy, there are still limitations to its direct application in vivo. First of all, NO is a double-edged sword gas which means that NO could promote the growth of cancer cells at low concentrations (<nM level) mediating antioxidants, signaling and positive bioenergetic mechanisms, while effectively killing high concentrations (≥nM level) of cancer cells by inhibiting mitochondrial respiratory metabolism [52,53]. It should be noted that a previous study found that NO causes blood poisoning above the concentration of 25 ppm in gas inhalation concentration [5]. Therefore, only in a certain range of concentration could NO exert an effective anti-tumor effect [54,55,56]. Secondly, the half-life of NO in vivo is very short, and it is difficult for the concentration of free NO to reach desired levels in tumor tissue [57]. In addition, NO exhibited nonspecific distribution in vivo, leading to its limited therapeutic effect and severe side effects [56]. Therefore, achieving the on-demand release of NO in tumor tissue is a key point to achieve the safe and effective treatment of tumors [56,58,59].

**Scheme 1:**
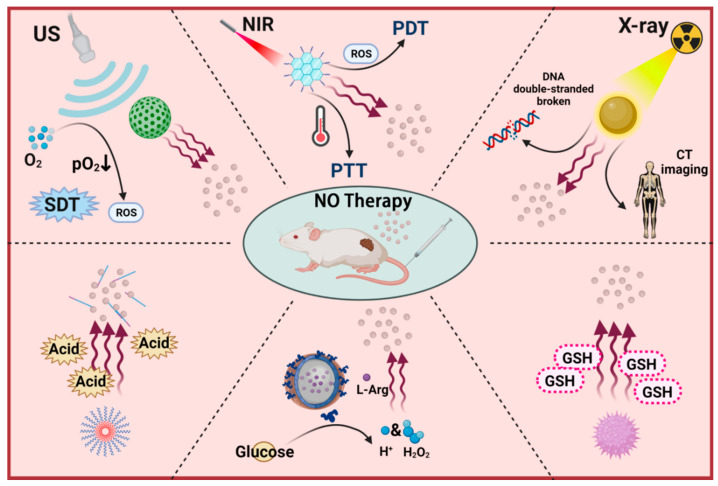
A review of the different endogenous and exogenous stimulus-responsive NO nanomedicines for the treatment of cancer. (Abbreviations—US: ultrasound; SDT: sonodynamic therapy; NIR: near infrared; PDT: photodynamic therapy; PTT: photothermal therapy; GSH: glutathione.)

In recent years, numerous studies have focused on the controlled release of NO by designing NO-based nanomedicines [60]. The release of NO could be controlled by an exogenous stimulus such as light, ultrasound and X-rays, which shows the characteristics of non-invasive and precise control with space–time selection. In addition, the TME has been found to be a critical foundation for tumor initiation and progression [61,62]. It exhibits unique pathological features such as a low oxygen level, low pH level, high-level glutathione (GSH), high-level glucose, high reducibility, high-level hydrogen peroxide (H_2_O_2_) and over-expressed enzymes [62,63,64,65]. Taking advantage of the TME, NO-based nanomedicines which could respond to low pH, high-level GSH and high-level glucose were developed [62,63,64]. The developed nanomedicines could remain stable in vitro or in normal tissues, which guaranteed the biosafety of the nanomedicines. Upon reaching the tumor(s), an exogenous or endogenous stimulus could trigger the on-demand release of NO which achieves the effective treatment of tumor. The most commonly used NO donors are N-Diazeniumdiolates (NONOates), S-Nitrosothiols (RSNOs), metal–nitrosyl complexes (such as Roussin’s black salt, red salt dianinion and ruthenium-nitrosyl) and nitrobenzene derivatives (such as *N*,*N*′-di-sec-butyl-*N*,*N*′-dinitroso-1,4-phenylenediamine, BNN6) [66]. Diverse NO donors usually exhibit different characteristics and properties. For example, NONOates can achieve the rapid release of NO gas under acidic conditions, but they are also not very stable under physiological conditions, leading to non-negligible premature leakage; RSNOs are relatively stable under physiological conditions while chemical bonds’ breakage and NO release can be achieved under hyperthermia [67]. Based on the advantages and disadvantages of different NO donors, many new responsive NO-releasing nanomedicines have been developed, as shown in Table 1. This review summarizes the design principles and release mechanisms of NO-based nanomedicines upon various stimuli and their applications in synergistic cancer therapy—as shown in Scheme 1.

## 2. Exogenous Stimuli-Responsive NO Nanomedicines

Since the exogenous stimuli can be conveniently controlled in vitro, it is advantageous for the on-demand release of NO-based nanomedicines in a spatio-temporal manner [73,106]. Therefore, numerous studies have reported NO-based nanomedicines controlled by exogenous stimulus in recent years [107,108]. External stimuli including light, ultrasound and X-rays provide a promising means of implementing the controlled release of NO which has achieved effective anti-tumor effects.

### 2.1. Light-Triggered NO Nanomedicines

#### 2.1.1. Ultraviolet–Visible Triggered NO Nanomedicines

**Figure 1:**
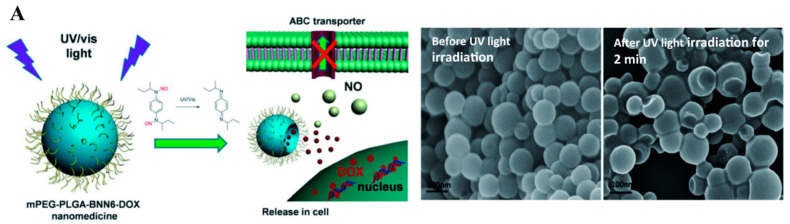

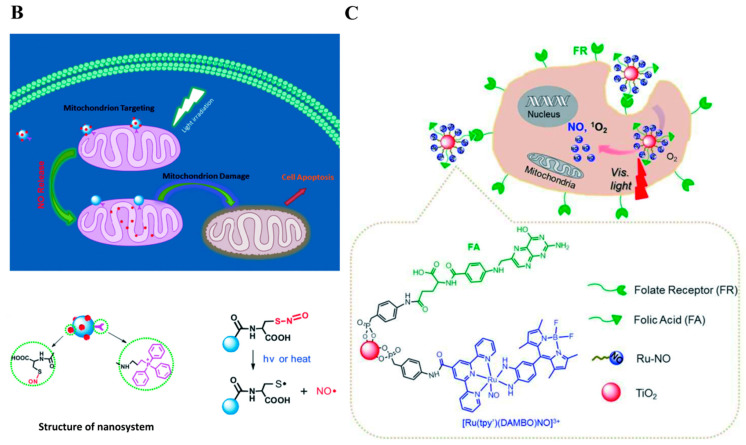
(**A**) Schematic diagram of NO release in cells by the mPEG–PLGA–BNN6–DOX nanosystem and the SEM images of the nanoparticles. Adapted with permission from [68], ACS Applied Materials & Interfaces, 2016; (**B**) schematic diagram of Cdot–TPP–SNO nanosystem releasing NO in cancer cells for anti-tumor therapy. Reproduced with permission from [69], Small, 2014; (**C**) schematic diagram of the NO release in tumor cells by the Ru–NO@TiO_2_NPs nanosystem. Reproduced with permission from [70], Chemical Communications, 2015.

Due to their non-invasive, practical, inexpensive and easily controllable nature, light has received significant attention as an exogenous stimulus in biomedical applications [109,110,111,112]. It has been reported that a large part of the light-sensitive NO donors could release NO in a controlled manner with the irradiation of ultraviolet–visible (UV–vis) light [113,114,115]. For example, a type of NO donor, named *N*,*N*′-Di-sec-butyl-*N*,*N*′-dinitroso-1,4-phenylenediamine (BNN6), can achieve the controlled release of NO under UV/vis irradiation. However, the hydrophobicity and toxicity of BNN6 hinder its direct biological application [82]. In view of this, Fan et al. encapsulated BNN6 with block polymer mPEG-PLGA, which exhibited superior aqueous dispersibility and stability. The obtained nanosystem could achieve the on-demand release of NO under UV–vis irradiation. The scanning electron microscopy (SEM) image indicated that the nanomedicine showed a uniform sperm shape before UV light irradiation and a collapsed state after UV light irradiation for 2 min. Meanwhile, NO was simultaneously produced, which effectively reverses the MDR of tumor cells by inhibiting the expression of P-gp on tumor cells (Figure 1A) [68]. Given that mitochondria are an important target for anti-tumor therapy, Wu et al. designed a mitochondrial-targeting photo-responsive NO-releasing nanomedicine using carbon dots (C-dots) as the carrier loaded with NO donor S-nitrosothiol (R-SNO) derivatives. It was found that the obtained nanomedicine was able to target mitochondria and release NO in response to the irradiation by UV–vis laser, which specifically damaged the mitochondria in tumor cell (Figure 1B) [69]. Furthermore, Liu et al. designed a folate-modified NO-based nanomedicine using metal nitrosyls Ru-NO as the NO donor. It was found that the nanomedicine could target human cervical HeLa cells that over-expressed folate receptors and released NO as well as singlet oxygen in response to UV–vis stimulation, resulting in a synergistic anti-tumor effect (Figure 1C) [70].

In addition to its therapeutic effect on tumors, NO has also shown unpredictable potential in the treatment of other diseases [66]. For example, as a gaseous signaling molecule, NO has been demonstrated to facilitate the processes of wound healing [116]. NO is involved in vascular dilation and anti-platelet activity during inflammation, which can promote angiogenesis at the stage of proliferation and collagen deposition at the stage of remodeling. Therefore, Duan et al. designed a nanosystem by directly polymerizing an NO donor: n-nitrosoamine-based monomers and wrapping them in a vesicle bilayer structure. The nanosystem could accurately release NO when stimulated by 365 nm UV, which could promote wound healing in a corneal wound model [117].

#### 2.1.2. First Near-Infrared (NIR-I) Photothermal Triggered NO Nanomedicines

Although many advances have been achieved in the UV–vis light-triggered NO releasing system, the low tissue penetration and high phototoxicity of UV–vis light limit its application in the field of anti-tumor therapy [118]. Compared with UV–vis light, NIR light has become an emerging hotspot because of its high tissue penetration and low photoxicity [53,119,120,121]. NIR can not only stimulate the release of NO from photo-sensitive NO donors but also mediate the role of photosensitizers in phototherapy, including photothermal therapy (PTT) and photodynamic therapy (PDT) [81,122,123]. PTT refers to the use of photosensitive agents to absorb NIR and convert it into heat to kill tumor cells [124]. Therefore, the use of photosensitive NO donors can effectively combine PTT and NO to play a synergistic anti-tumor role [125,126,127,128]. Depending on the wavelength, NIR could be divided into two categories: first NIR window (650–900 nm) and second NIR window (900–1200 nm). NIR-I light has been the most used NIR light in the treatment of tumors in previous studies. In this section, NIR-I light triggered NO nanomedicines are introduced and analyzed.

**Figure 2:**
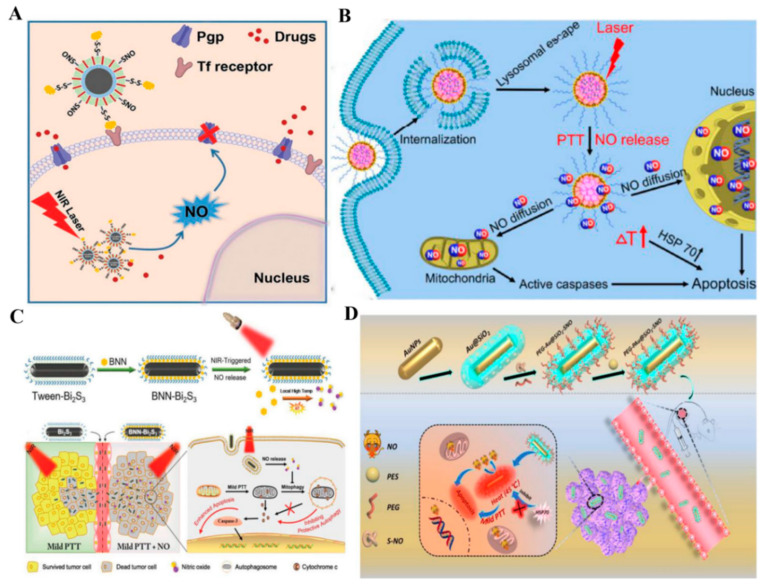
(**A**) Schematic diagram of the preparation and release of NO from photo-triggered NO nanogenerators (PTNG). Reproduced with permission from [71], Advanced Functional Materials, 2017; (**B**) schematic diagram representation of the therapeutic function of PpRE@PEG–PpIX NPs. Reproduced with permission from [72], Nano Research, 2019; (**C**) schematic diagram of the synthesis and NIR light-triggered NO release from the BNN–Bi_2_S_3._ Reproduced with permission from [74], Advanced Science, 2019; (**D**) schematic diagram of the preparation of PEG–PAu@SiO_2_–SNO and the process of mild heat-enhanced NO therapy under NIR irradiation in cancer cells. Reproduced with permission from [76], ACS Biomaterials Science & Engineering, 2020.

It has been reported that MDR is an important reason for chemotherapy failure [129,130]. Thus, Yang et al. used Fe_3_O_4_@polydopamine@mSiO_2_ as a photothermal carrier to prepare NIR light-triggered nanomedicine. By receiving NIR stimulation and converting NIR light into heat, the nanomedicine could break the chemical bond of R-SNO and achieve the controlled release of NO, which could reverse the MDR and realize the combined treatment of tumor with NO therapy, chemotherapy and PTT (Figure 2A) [71]. To further enhance the light absorption rate and photothermal conversion efficiency, Huang et al. used intelligent protoporphyrin with high light absorptivity as a photosensitizer to design a novel polymer nanomedicine. The nanomedicine could efficiently release NO under the irradiation of NIR, which could effectively kill tumor cells with low laser power density (Figure 2B) [72]. Furthermore, Liu et al. designed a novel multifunctional NIR-responsive magnetic nanoplatform, which can be guided by a magnetic field to achieve high nanoparticle accumulation in tumor tissue. Under the irradiation of an 808 nm laser, the photothermal effects led to the on-demand release of NO, which achieved remarkable anti-tumor effects [73].

In the process of PTT, high temperature is needed to effectively kill tumor cells, but it will inevitably cause damage to the surrounding normal tissues. In order to prevent this, researchers have tried to use a mild photothermal therapy (mPTT) strategy to solve this dilemma, which, however, can easily lead to the self-repair of tumor cells after insufficient heat damage [131]. In this context, Zhao et al. designed a nanocomposite using bismuth sulfide (Bi_2_S_3_) nanoparticles as a photothermal carrier loaded with an NO donor. The obtained nanocomposite could achieve the mPTT effect under the irradiation of NIR laser, which led to the controlled release of NO, thereby improving the anti-tumor effect of mPTT (Figure 2C) [74]. Furthermore, Dong et al. designed an NIR responsive nanocomposite platform to combine the mPTT with gas and chemotherapy. Under the stimulation of the NIR laser, the thermosensitive NO donor achieved the on-demand release of NO, which could overcome the MDR of tumor cells and enhanced the chemotherapy effect of doxorubicin (DOX). At the same time, NO and chemotherapy could effectively compensate for the therapeutic deficiency of mPTT, achieving a combined anti-tumor effect [75]. With the further exploration of mPTT in tumor therapy, studies have found that cunning tumor cells produced a series of heat shock proteins, which protected tumor cells from damage at low heat levels [132]. Therefore, Wang et al. encapsulated heat shock protein 70 (HSP70) inhibitor 2-phenylethyne sulfonamide (PES) into the NO-based nanomedicine. The mild heat generated by the nanomedicine under the irradiation of NIR could not only induce cell apoptosis with the assistance of PES at a relatively low temperature, but also released NO, thus achieving the synergistic effect of mPTT and NO therapy (Figure 2D) [76].

#### 2.1.3. Second NIR (NIR-II) Photothermal Triggered NO Nanomedicines

**Figure 3:**
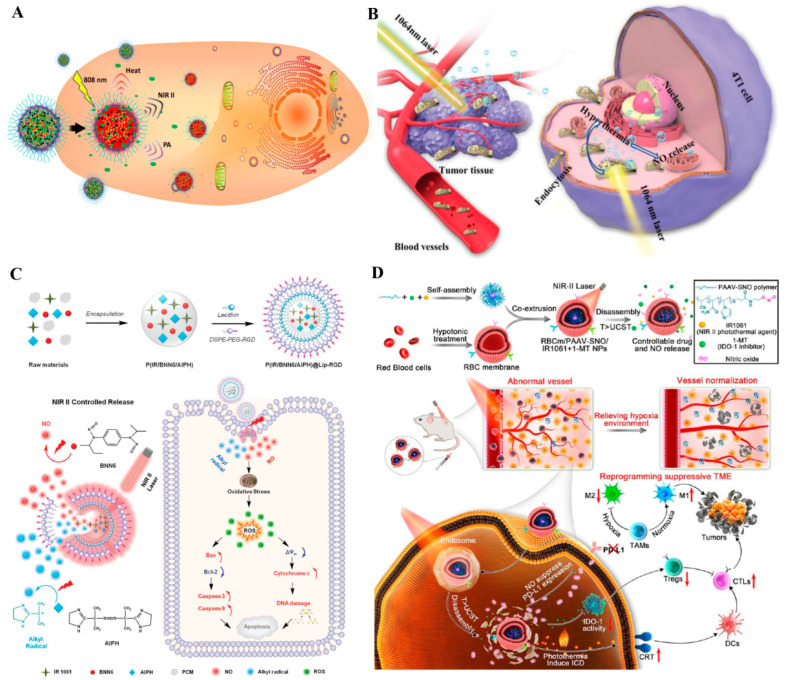
(**A**) Schematic diagram of the PFTDPP–SNAP which achieved PTT and NO therapy with NIR light irradiation. Reproduced with permission from [77], Nano Letters, 2019; (**B**) schematic diagram representation of the therapeutic function of Nb_2_C–MSNS–SNO. Reproduced with permission from [33], Advanced Science, 2020; (**C**) schematic diagram of preparation of P(IR/BNN6/AIPH)@Lip–RGD and the process of PTT and NO therapy with NIR-II irradiation in cancer cells. Reproduced with permission from [78], Nanoscale, 2021; (**D**) schematic diagram of the preparation and drugs releasing process of nanomedicine and the process of reprogramming tumor immunosuppressive microenvironment and fighting immune cold tumor. Reproduced with permission from [79], ACS Nano, 2020.

Compared with NIR-I light, NIR-II exhibited deeper tissue penetration depth and higher maximum skin exposure density (1.0 w cm^−2^), which has emerged as a research hotspot in recent years. For example, Fan et al. designed semiconductor polymer nanoparticles (PFTDPP) with spectral adjustability and electro-optical property. Under the irradiation of 808 nm laser, the nanomedicine could absorb NIR and emit NIR-II (1000–1700 nm) to achieve the accurate tumor location by NIR-II/photoacoustic (PA) imaging. At the same time, the photothermal effect could also stimulate the release of NO from the NO donor (S-nitroso-N-acetylpenicillamine, SNAP), which represented a combination of dual-mode imaging and NO therapy to achieve the better anti-tumor effect (Figure 3A) [77]. In addition to the first NIR biological window (NIR-I) (700–950 nm), the second NIR biological window (NIR-II) (1000–1700 nm) can also be used as the stimulus to trigger the release of NO [133,134,135,136]. Compared with NIR-I, NIR-II has a deeper penetration depth of mammalian tissue and higher maximum allowable skin exposure (MPE) [137,138]. For example, owing to the excellent adsorption ability of MXenes in the NIR-II window, Xu et al. designed a type of MXenes-based nanomedicine modified with the NO donor which could achieve the synergistic/sequential anti-tumor therapy [139,140,141]. MXenes could effectively convert NIR-II laser into heat to break SNO and released NO at high temperature, thus realizing the effective inhibition effect on the tumor(s) (Figure 3B) [33]. It has been found that alkyl radical, a type of free radical independent from oxygen, can damage lipid and DNA by increasing the oxidative stress of tumor cells. Thus, Xue et al. prepared a novel nanomedicine by encapsulating the NIR-II molecule (IR 1061), the NO donor BNN6 and alkyl radical initiator 2,2-azobis[2-(2-imidazolin-2-yl) propane] dihydrochloride (AIPH) in an amphiphilic lecithin stabilized phase change material with a eutectic point of 39 °C. The nanomedicine produced photothermal effects under NIR-II irradiation to release BNN6 and AIPH, which then decomposed to form highly active NO and alkyl radical. The synergistic action of NO and alkyl radicals induced the high oxidative stress in mitochondria, which could not only accelerate the release of cytochrome C to cause mitochondrial membrane damage but also significantly down-regulate the Bcl-2 protein to trigger the apoptosis of tumor cells. Animal experiments have shown that mice treated with this strategy can survive for more than 40 days and have a tumor suppression rate of more than 70% (Figure 3C) [78]. Furthermore, Tian et al. designed a type of microsphere for the treatment of cold tumors using the photothermal agent IR1061, NO donor SNO and 1-methyl-tryptophan(1-MT). The IR1061 could achieve PTT by converting the light energy of NIR-II laser into heat energy. PTT not only induced immunogenic cell death (ICD) to recruit CD8^+^ cytotoxic T lymphocytes (CTL), but also act as an external stimulus which accurately triggered NO release by breaking the S-NO bond with the action of heat. The released NO normalized the tumor blood vessels by inducing endogenous angiogenic factors, which alleviated the hypoxia of TME and reprogrammed the immunosuppressive TME by, e.g., decreasing the level of immunosuppressive cells (Tregs, M2-like macrophages) and the expression of immune checkpoints (PD-L1) [142]. Therefore, a therapeutic strategy that achieved the PTT-augmented NO therapy in response to NIR-II stimulation could effectively reverse the “cold tumor” into highly immunity-invasive “hot tumor” and reprogram the immunosuppressive TME into an immunostimulating phenotype. The in vivo experiments showed that, on day 21, four of the six tumor-bearing mice survived without significant weight loss. The survival rate of tumor-bearing mice reached 66.7% on day 48 after the treatment, with efficacy in both primary breast cancer and metastatic tumor (Figure 3D) [79].

#### 2.1.4. NIR Photodynamic Triggered NO Nanomedicines

**Figure 4:**
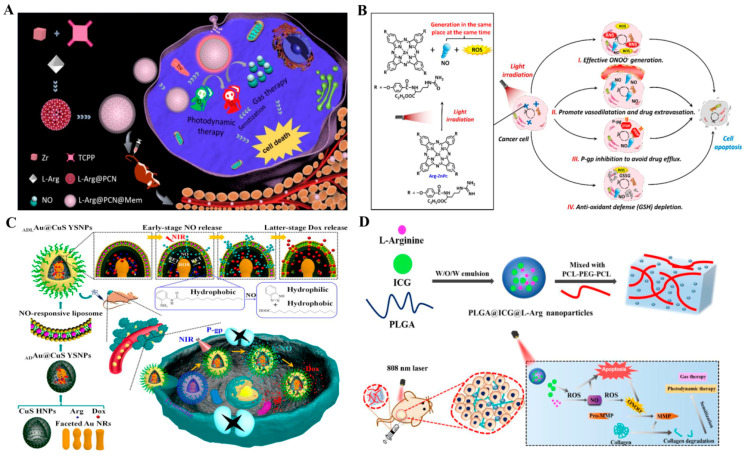
(**A**) Schematic diagram of the preparation of the L-Arg@PCN@Mem and the tumor-killing mechanism. Reproduced with permission from [83], Biomaterials, 2018; (**B**) mechanism diagram of cancer cells apoptosis facilitated by the integration of various functions within L-arginine ethyl ester-modified zinc phthalocyanine (Arg–ZnPc). Reproduced with permission from [84], Molecular Pharmaceutics, 2020; (**C**) schematic diagram of the preparation of L-arginine/Dox-loaded gold@copper sulfide yolk−shell nanoparticles (ADLAu@CuS YSNPs), the mechanism diagram of the process of NO and DOX being sequentially released plays an anti-tumor effect under NIR irradiation in cancer cells. Reproduced with permission from [85], Nano Letters, 2019; (**D**) schematic diagram of the preparation of PLGA@ICG@L-Arg nanoparticles and the process of anti-tumor effects under NIR irradiation in cancer cells. Reproduced with permission from [86], Molecular Pharmaceutics, 2021.

In addition to PTT, PDT is also an effective approach to trigger the release of NO [143]. PDT involves NIR stimulating the photosensitive material, which transfers energy to the surrounding tissues to produce a large number of reactive ROS [144,145]. For example, Xiang et al. designed a dual-targeting nanoplatform to target the lysosomal organelles in the folate receptor over-expressed cancer cells. NO and ROS were released under the NIR irradiation, exerting the synergistic anti-tumor effect of gas therapy and PDT [87]. Among the conventional targeted nanoplatforms that have been reported, there are often challenges such as difficulty in synthesis and the potential immune response of the modified ligands [146,147,148]. Therefore, based on the abundance antigens on the surface of cell membrane, Zhang et al. prepared a porous coordination network that was coated with the cell membrane of the murine mammary carcinoma 4T1 cells. This nanosystem could achieve the effective tumor accumulation and negligible immune response. Under the stimulation of the NIR laser, a large amount of ROS could be directly generated to realize the PDT in tumor tissue. ROS generated by PDT could further react with NO to produce more toxic reactive nitrogen (RNS), which represents one of the anti-tumor mechanisms of NO (Figure 4A) [83]. Wei et al. synthesized a novel multifunctional nanosystem by integrating the NO donor into a photosensitizer. The nanosystem releases NO under NIR laser irradiation, which could promote vascular dilatation and overcome the MDR of tumor cells. In addition, the released ROS by PDT could react with NO, which ensured the generation efficiency of RNS with high toxicity. In addition, NO could also destroy the antioxidant system of tumor cells by consuming high concentrations of GSH in tumor cells, which further improved the efficiency of oxidative damage of RNS. This multifunctional synergistic effect could effectively guarantee the anti-tumor therapeutic effect (Figure 4B) [84]. In order to further improve the anti-tumor effect, Wang et al. designed a nanosystem to sequentially release the loaded drugs. Under the stimulation of the NIR laser, the nanosystem absorbed the laser to produce ROS, which could oxidize the NO donor L-Arginine (L-Arg) to produce NO. When NO gradually increased, the liposome layer of the nanosystem was gradually destroyed and followed by the release of DOX. The sequential release of NO and DOX could firstly enable NO to reverse MDR and provided a favorable time window and cellular environment for the anti-tumor effect of DOX (Figure 4C) [85]. In addition, hydrogels, as carriers with low toxicity, good biocompatibility, local drug delivery and slow release, are increasingly being applied in anti-tumor gas therapy [149]. For example, Sun et al. designed a responsive hydrogel using indocyanine green (ICG) as the photosensitizer and L-Arg as the NO donor. In 4T1 tumor-bearing mice, the obtained hydrogel was locally injected into tumors tissues, where ROS and NO could be produced in situ under NIR irradiation to exert synergistic anti-tumor effects. Meanwhile, studies have shown that components such as collagen or hyaluronic acid, which are highly expressed in the extracellular matrix of tumor, could block the entry of nanoparticles and immune cells into solid tumors and weaken the anti-tumor effect. The matrix metalloproteinases (MMPs) could effectively degrade tumor extracellular matrix and improve the penetration of nanoparticles and immune cells [150,151,152]. Therefore, the RNS generated by ROS and NO could activate MMPs in tumor cells which could further degrade the collagen in the extracellular matrix, which modulated the TME, leading to the improved drug sensitivity and immunotherapy. In animal experiments, the bioluminescent imaging signal intensity in mice treated with this method showed a significant increase in the efficiency of tumor growth inhibition of up to 60% (Figure 4D) [86].

Additionally, osteoporosis, a bone metabolic disease with a high risk of fracture, has been found to be reversed by NO which could promote the proliferation and differentiation of osteoblasts. Therefore, Ye et al. developed a bone-targeted upconversion nanoparticles (UCNP) by loading modified bisphosphonate—alendronate (Ald)—onto a mesoporous silica surface followed by an NO donor—BNN6. Under the irradiation of 808 nm NIR, the nanosystem could achieve the precise release of NO in the bone, thereby promoting osteoblast formation and ultimately reversing osteoporosis [153].

### 2.2. Ultrasound Triggered NO Nanomedicines

**Figure 5:**
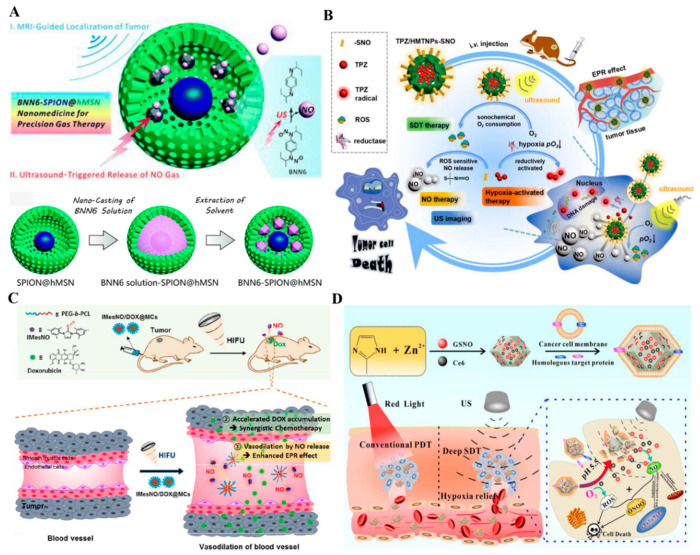
(**A**) Schematic diagram of the preparation of BNN6–SPION@hMSN which could achieve the controlled release of NO with ultrasound stimulation. Reproduced with permission from [88], Nanoscale, 2017; (**B**) schematic diagram of the therapeutic modes of TPZ/HMTNPs–SNO in cancer cells. Reproduced with permission from [89], Journal of Controlled Release, 2018; (**C**) Schematic diagram of the anti-tumor effect of nanomicelles IMesNO/DOX@MCs under HIFU irradiation. Reproduced with permission from [90], Biomaterials, 2019; (**D**) schematic diagram of the preparation of the GSNO/Ce6@ZIF-8@Cytomembrane (GCZ@M) and the process of anti-tumor effects in cells. Reproduced with permission from [91], Biomaterials, 2019.

Compared to other stimuli, ultrasound has the advantage of deeper tissue penetration with a maximum depth reaching 20 cm at the power density of 1 MHz [154]. In addition, ultrasound exhibited the characteristics of non-invasiveness, easy to control and easy to focus on a particular area. Therefore, ultrasound responsive nanomedicines are good candidates for the controlled release of NO [155,156,157]. In the drug delivery process, it was found that the intercellular matrix hindered the efficient transport of nanomedicines to tumor tissues [158]. Therefore, Chen et al. designed ultrasonic-responsive nanosystems modified with cyclic decapeptides which can target fibrinogen and achieve the ‘relay’ transmission of the nanomedicines from the blood vessel to the stroma and then to the tumor cells, which increased the retention rate of the nanomedicines in tumor cells by almost seven times. Under the local ultrasound stimulation, the responsive release of NO from the nanomedicines could be achieved, which played a significant anti-tumor effect [93]. In addition, in order to accurately target the tumor site, He et al. designed an ultrasonic-triggered nanomedicine using paramagnetic iron oxide nanoparticles with MRI imaging guidance functions. The developed nanomedicine could be clearly localized under the MRI guidance and released NO under ultrasonic stimulation to achieve efficient and safe tumor therapy (Figure 5A) [88]. In addition, ultrasonic responsive nanomedicines can also be used in combination with sonodynamic therapy (SDT) to treat tumors. For example, Feng et al. designed a novel ultrasonic-responsive sequential nanomedicine using hollow mesoporous titanium dioxide nanoparticles loaded with anoxic active prodrug tirapazamine (TPZ) and SNO. Under the stimulation of ultrasound, the nanosystem generated ROS through SDT, which caused the breakage of SNO and released NO in a controlled manner. With the continuous function of SDT, the hypoxic environment formed by the consumption of oxygen could further effectively activate TPZ to lyse the DNA in tumor cells. The ultrasonic-responsive sequential SDT/NO therapy could effectively enhance the anti-tumor effect (Figure 5B) [89]. High-intensity focused ultrasound (HIFU) therapy can kill tumor cells by forming high temperature in the target area through the thermal effect at the focus. Kang et al. combined the thermal effect of HIFU with ultrasound responsive nanosystem to strengthen the anti-tumor effect. Under the action of HIFU, ultrasound converged to the tumor site to produce high temperature, resulting in tumor tissue degeneration and necrosis. Meanwhile, NO was effectively released at high temperature to dilate the blood vessels at the tumor site, thus promoting the effective accumulation of the injected nanomedicines (Figure 5C) [90]. In order to enable the nanosystem to target tumor cells more effectively, Zhao et al. took advantage of the homologous targeting of the tumor cell membrane by encasing NO donor GSNO and dihydroporphyrin e6 (Ce6) in the tumor cell membrane, and finally designed a bionic nanosystem with both pH and ultrasonic response. Under the action of the tumor cell membrane, the nanosystem could effectively target to the tumor tissue. In the tumor acidic environment, GSNO and Ce6 were released with the stimulation of exogenous ultrasound. In addition, ROS produced by Ce6 under ultrasound could interact with NO to produce more toxic RNS to kill tumor cells. This work is the first example to realize gas-sonodynamic combined therapy for anti-tumor therapy, thus opening a new avenue for ultrasound-triggered gas therapy (Figure 5D) [91]. In addition, platelets are known to maintain vascular integrity in tumors, whose function could be inhibited by NO. Xu et al. loaded the chemotherapy drug paclitaxel (PTX) into an albumin shell modified with the NO donor SNO, which designed an NO-based nanosystem, SNO–HSA–PTX. The nanosystem was able to respond to ultrasound stimulation which triggered the release of NO. Therefore, the function of tumor-associated platelets was inhibited, which induces the opening of the tumor vascular barrier and promotes the accumulation of PTX and immune cell invasion. Through the combination of chemotherapy, immunotherapy and NO gas therapy, remarkable tumor suppression was finally achieved with negligible side effects [92].

### 2.3. X-ray-Triggered NO Nanomedicines

**Figure 6:**
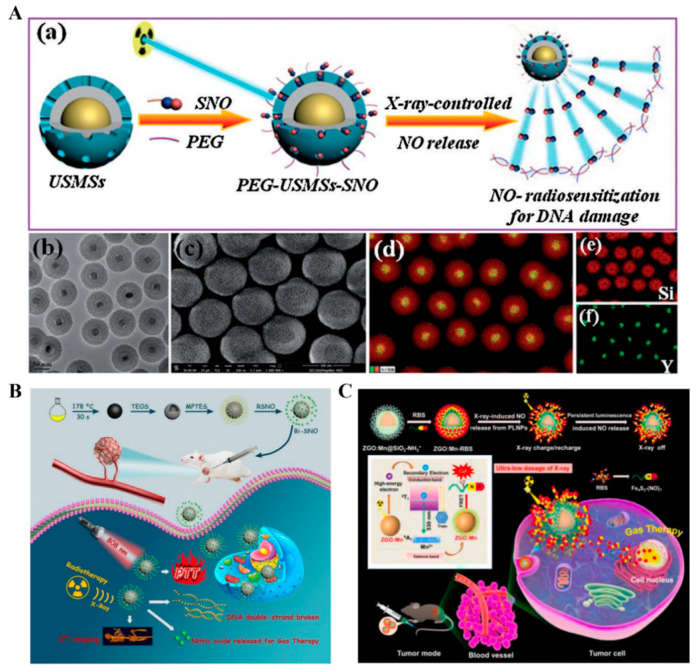
(**A**) (**a**) Schematic diagram of PEG–USMSs–SNO construction; (**b**) transmission electron microscope images of USMSs; (**c**) SEM images of USMSs; (**d**) elemental mapping of the merging of Si and Y of USMSs; (**e**) elemental mapping of merging of Si of USMSs; (**f**) elemental mapping of merging of Y of USMSs (USMSs: a dense silica shell was coated on upconversion nanoparticles). Reproduced with permission from [94], Angewandte Chemie International Edition, 2015. (**B**) schematic diagram of the preparation of Bi–SNO-NPs and the process of enhanced NO therapy with X-ray irradiation in cancer cells. Reproduced with permission from [95], Nanoscale, 2020. (**C**) schematic diagram of the preparation of the ZGO:Mn-RBS nanosystem and the process of anti-tumor effects. Reproduced with permission from [96], Biomaterials, 2020.

X-rays, as an exogenous excitation source, have been widely used in imaging and cancer therapy in clinics. In recent years, X-rays have also been used as exogenous stimulation to achieve the controlled release of NO [159]. For example, Shi et al. successfully constructed intelligent X-ray-responsive upconversion nanosystems which could track and monitor the drug delivery and release process under the radiation of X-rays. Meanwhile, the water in the tumor could be decomposed into ROS under the X-rays irradiation, which reacted with SNO to trigger the release of NO, thereby achieving the combined tumor treatment (Figure 6A) [94]. Additionally, bismuth nanoparticles with high X-ray absorption coefficients can also be used to track the delivery process of the NO-based nanomedicines in real time [160]. For example, Zhang et al. used bismuth-based nanoparticles to design a multifunctional X-ray responsive nanosystem, which could be used for computed tomography (CT) imaging under X-ray radiation. Besides, the chemical bond of SNO was broken, leading to the controlled release of NO with X-ray irradiation. In addition to directly killing tumor cells, a high concentration of NO could also enhance the radiotherapy effect through Compton scattering and Auger effect, which synergistically enhanced the anti-tumor effect (Figure 6B) [95]. Although numerous progress has been made in X-ray responsive nanosystems, most of the previously reported X-ray responsive NO releasing nanosystems requires high X-ray doses to break the chemical bond of the NO donor. In this context, Hao et al. designed a novel soft X-ray responsive continuous luminescent release nanosystem by using persistent luminescent nanoparticles (PLNPs) as the carrier. When irradiated by soft X-rays (doses as low as 0.9 mGy), the nanosystem stored energy through PLNPs which continuously released NO in deep tumor tissues (up to 20 mm deep). The nanosystem continued to release NO even 40 min after the cessation of X-ray irradiation (Figure 6C) [96]. This complementary therapy strategy of the X-ray and NO brings a broader idea for tumor therapy and becomes one of the key points of anti-tumor research on stimuli-responsive NO nanomedicines in the future [161,162,163,164,165].

## 3. Endogenous Stimuli-Responsive NO Nanomedicines

As is already known, TME is characterized by low pH, high-level glucose, high-level GSH, high-level H_2_O_2_ and over-expressed enzymes, etc. [166,167,168,169]. Therefore, many researchers have designed endogenous responsive NO release nanomedicines taking advantage of these characteristics [106]. Compared to exogenous stimuli, endogenous stimuli are not limited by tissue depth and do not require external equipment, so they can reduce treatment costs and significantly improve the compliance of the patients. In addition, nanomedicines can be spontaneously activated at the tumor site under the action of endogenous stimulation to achieve precisely controlled NO release [170,171]. Therefore, endogenous stimulations, including low pH, high-level glucose and high-level GSH, also provide a promising pathway for the controlled release of NO in vivo.

### 3.1. Glutathione-Triggered NO Nanomedicines

**Figure 7:**
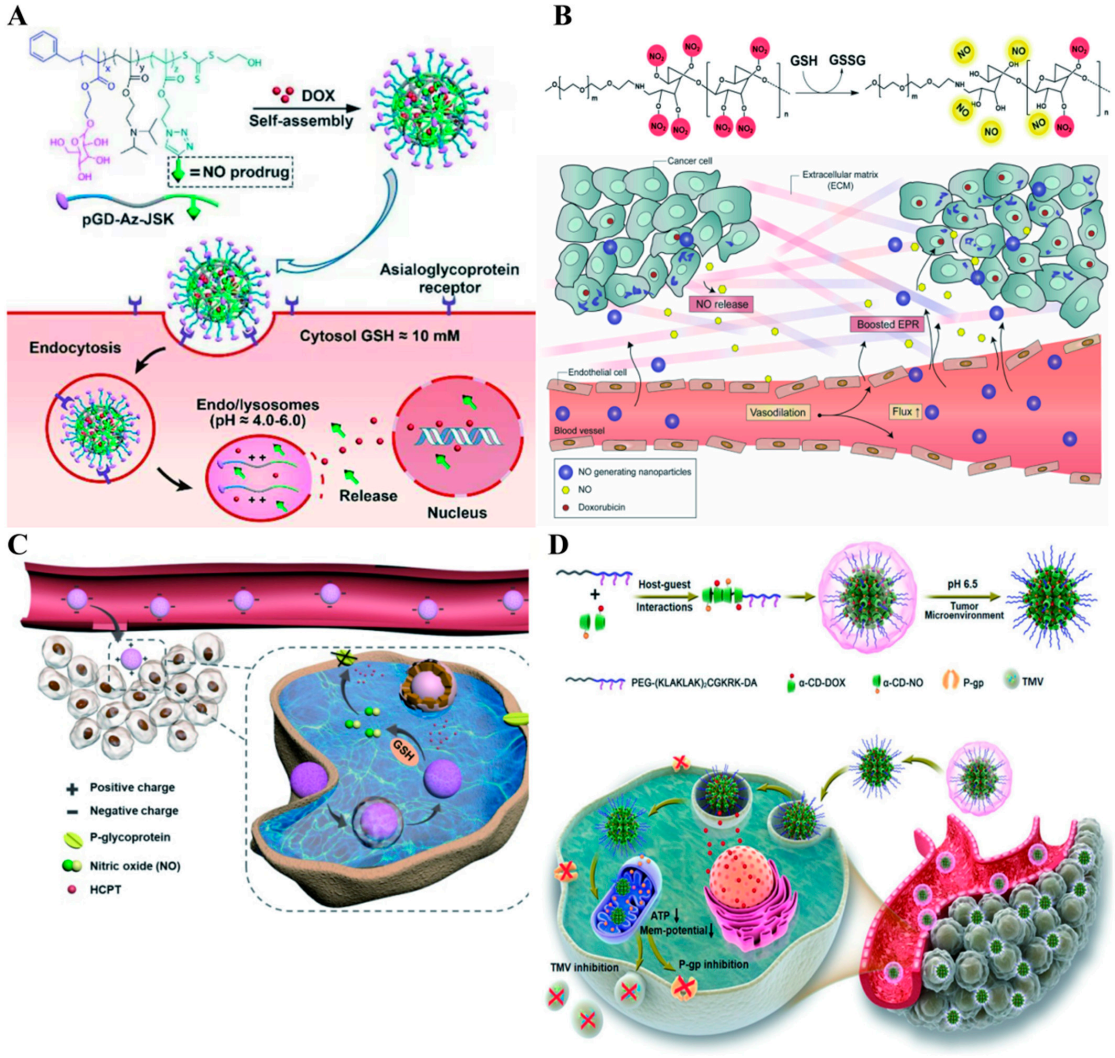
(**A**) Schematic diagram of the structure and preparation of the p(Gd-Az-JSK) NPs and their delivery process. Reproduced with permission from [97], Nanoscale, 2018; (**B**) schematic diagram of PEG-b-NO-Dex nanoparticles releasing NO and boosting EPR in the presence of GSH. Reproduced with permission from [98], Nano Letters, 2018; (**C**) schematic diagram of the structure and preparation of the HCPT/CTS–NO–DMMA NPs and its delivery process. Reproduced with permission from [99], Nanomedicine, 2019; and (**D**) schematic diagram of preparation of the α-CD–DOXNO–DA NPs and the process of exerting anti-tumor effects in cancer cells. Reproduced with permission from [100], Small, 2020.

As is already well-known, the TME is characterized by high GSH with concentration 7–10 times higher than that of normal tissues. Therefore, GSH could act as an endogenous stimulus to trigger the release of NO [172,173]. Song et al. previously synthesized a new type of NO donor, named a nitrate-functionalized D-α-tocopheryl polyethylene glycol succinate (TNO_3_). It was found that TNO_3_ could chemically react with a high concentration of GSH in TME to achieve the controlled release of NO. The accumulation of NO in tumor tissues could enhance the EPR effect and increase the accumulation of DOX in tumor sites, thus improving the efficacy of chemotherapy [101]. Zhang et al. designed a novel nanosystem which encapsulated the NO donor O_2_-(2,4-dinitrophenyl) 1-[4(propargyloxycarbonyl) piperazin-1-yl] diazen-1-ium-1,2-diolate (alkynyl-JSK) in an amphiphilic block copolymer via a click reaction. After entry into tumor cells, alkynyl-JSK locally generated NO through a nucleophilic reaction with GSH. Especially, when this nanosystem is co-treated in combination with DOX, the anti-tumor efficacy can be further enhanced through a synergistic effect (Figure 7A) [97]. Moreover, Park et al. used a hydrophobic nitrifying glucan (NO-Dex) as the NO donor, and encapsulated it in hydrophilic PEG to obtain a new type of nanomedicine. After entering the tumor site, NO is produced by nitrate reduction reaction between GSH and NO-Dex. The release of NO could dilate blood vessels, increase vascular permeability, and promote the positive feedback effect of EPR (Figure 7B) [98]. Furthermore, Yuan et al. used the chemotherapeutic drug hydroxycamptothecin to prepare 10-hydroxycamptothecin-loaded chitosan nanoparticles (HCPT) by the ionic gel and chemical crosslinking method. The NO donor in the nanosystem could effectively release NO with the stimulation of GSH. It was found that NO could nitrate some tyrosine residues of P-gp on the surface of tumor cell membrane, thus inhibiting the pump function and enhancing the effective accumulation of chemotherapeutic drugs in tumor cells (Figure 7C) [99]. Moreover, Deng et al. found that tumor-derived microvesicles (TMVs), which are rich in proteins, lipids and RNA, promoted tumor progression and metastasis by regulating TME. The energy required for the production and release of TMV from tumor cells was closely related to mitochondria. Therefore, they designed a nanosystem capable of delivering SNO to mitochondria for targeting the release of NO. The amide bond in the nanosystem was hydrolyzed in the acidic TME and selectively targeted the mitochondria, which enabled the NO donor to accumulate in the mitochondria. Studies have shown that GSH can react with the NO donor to generate NO and directly cut off the mitochondrial energy supply, thereby effectively inhibiting TMV and thus preventing cancer metastasis (Figure 7D) [100].

### 3.2. pH-Triggered NO Nanomedicines

**Figure 8:**
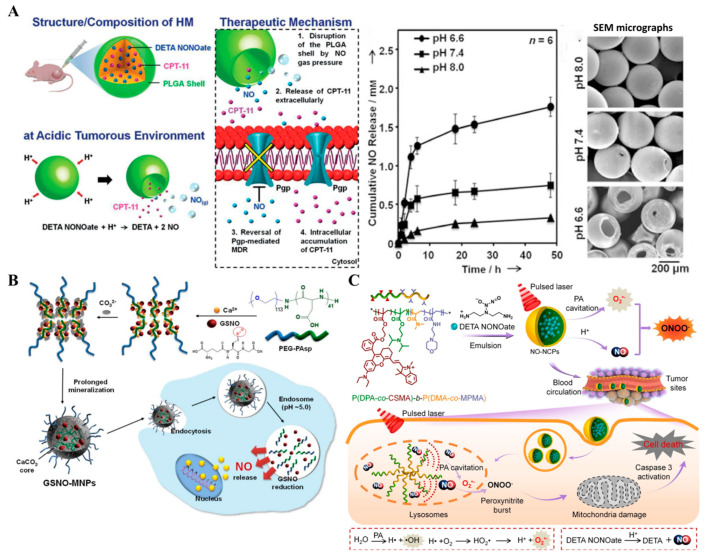
(**A**) Schematic structure/composition of hollow microspheres (HMs) and the mechanism in the treatment of tumors. Reproduced with permission from [49], Angewandte Chemie International Edition, 2015; (**B**) schematic diagram of the preparation of the GSNO–MNPs and the intracellular NO release from the GSNO-loaded CaCO_3_-mineralized nanoparticles (GSNO-MNPs). Reproduced with permission from [102], Colloids and Surfaces B: Biointerfaces, 2016; and (**C**) schematic diagram of the preparation of the NO-based nanosystem. Reproduced with permission from [103], Angewandte Chemie International Edition, 2021.

Tumor cells produce a large amount of lactic acid because of their rapid growth and enhanced glycolysis metabolism, leading to the weakly acidic TME (pH 6.5~6.8) [174,175]. Therefore, an increasingly number of studies have taken advantage of the acidic TME and designed different pH-responsive NO-releasing nanomedicines based on it in recent years [173,176,177,178]. For example, Sung et al. designed a pH-sensitive release nanosystem loaded with chemotherapeutic drugs and the NO donor diethylenetriamine diazeniumdiolate (DETA NONOate). In the acidic environment of the tumor, H^+^ chemically reacts with DETA NONOate to release NO. The responsive release of NO could overcome the MDR and increase the accumulation of chemotherapeutic drugs, thereby reducing the survival rate of tumor cells (Figure 8A) [49]. In addition, based on the unique solution behavior of the biominerals’ calcium carbonate (CaCO_3_), Lee et al. designed a pH-responsive release nanosystem using CaCO_3_ as a carrier. When the nanosystem was internalized into tumor cells, acidic organelles (pH~5.0) dissolved CaCO_3_ and accelerated the release of NO donor GSNO. GSNO could react with reductive ascorbic acid in tumor cells to produce NO. Furthermore, the research group showed that the release of NO was much higher in the acidic pH environment than that in the neutral pH environment. It was found that a low pH is indeed a key factor to trigger the release of NO, which is expected to achieve the intracellular delivery of NO (Figure 8B) [102]. In addition, Wang et al. used the acid-responsive amphiphilic diblock copolymers to encapsulate the NO donor DETA NONOate. The obtained nanocapsules were found to be degraded in the acidic environment of tumor cell lysosomes which triggered the release of NO from DETA NONOate. More importantly, photoacoustic cavitation could stimulate ROS production and the formation water molecules in tumor cells under a pulsed laser. ROS could react with NO in situ to produce more toxic RNS, which could effectively inhibit the growth of tumor cells (Figure 8C) [103].

### 3.3. Glucose-Triggered NO Nanomedicines

**Figure 9:**
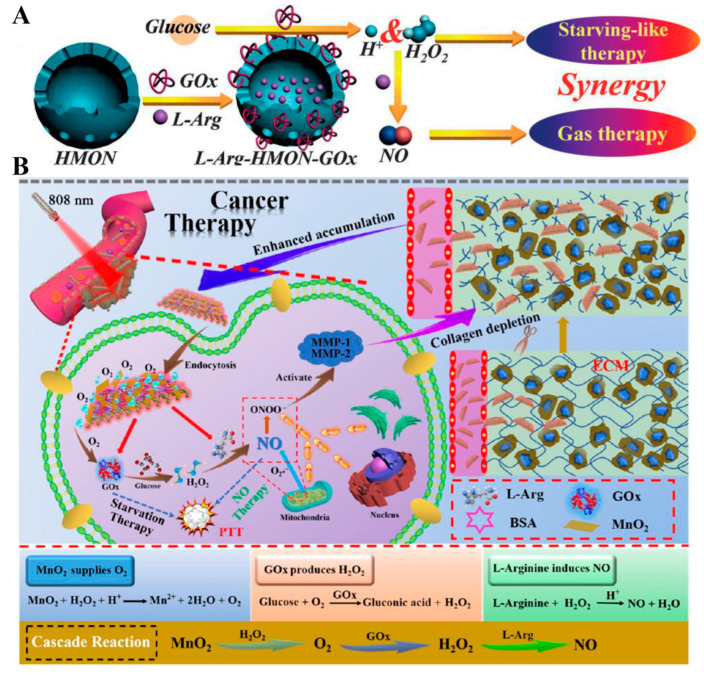
(**A**) Schematic diagram of the construction of L-Arg–HMON–GOx for synergistic cancer therapy. Reproduced with permission from [104], Angewandte Chemie International Edition, 2017; and (**B**) schematic diagram of the preparation of the BPNs–Arg–GOx@MnO_2_ (BAGN) nanosystem and the intracellular NO release. Reproduced with permission from [105], ACS Applied Materials & Interfaces, 2021.

It is well known that tumors require more nutrients and energy than normal tissues to maintain growth, leading to the higher concentration of glucose in the TME than in normal tissue [177,179]. Taking advantage of this, Fan et al. designed a novel glucose-responsive NO release nanosystem by loading glucose oxidase (GOx) and L-Arg in hollow silicone nanoparticles (HMON). The GOx in the nanosystem could consume glucose in the TME and inhibit tumor cell growth by glucose consumption (starvation therapy). On the other hand, H_2_O_2_ produced by glucose consumption could oxidize NO donor L-Arg into NO, which subsequently reacted with NO to generate more toxic peroxynitrite molecules to kill tumor cells. Using U87MG cells as target cells, the researchers found that with the increase in glucose concentration in the cell medium, the release of NO from the cells increased as well. In addition, the activity of tumor cells was significantly reduced when the nanosystem was used in a high concentration glucose medium. The results of the in vitro experiment proved that endogenous glucose in tumor cells could effectively stimulate the release of NO. Meanwhile, GOx-triggered starvation therapy could further synergistically kill tumor cells. In the U87 tumor-bearing mice, it was observed that the mice treated with the nanosystem had significantly smaller tumors than the mice treated with the other groups. This further confirmed the remarkable therapeutic effect of starvation therapy combined with the release of NO under the stimulation of glucose in tumors (Figure 9A) [104]. Recently, Su et al. innovatively designed a multimodal nanosystem for collaborative tumor treatment, which also used glucose-consuming starvation therapy to fight tumors. They used black phosphorus nanosheets (BPNs) with high photothermal conversion efficiency as carriers which loaded with NO donor L-Arg and GOx to obtain BPNs–Arg–GOx. BPNs–Arg–GOx was further modified with manganese dioxide nanosheets (MN) to obtain a nanosystem (BAGN). When the nanosystem entered the solid tumor site, it reacted with glucose to generate H_2_O_2_ which could promote the oxidation response of NO donor L-Arg to release NO. The NO produced by the nanosystem degrades the extracellular matrix in solid tumors by up-regulating the expression of MMP, which facilitated the accumulation of the nanosystem in the tumor site. In addition, the nanosystem could decompose H_2_O_2_ to produce oxygen to alleviate the hypoxia of solid tumors. Manganese ion and oxygen also provided good T1-weighted magnetic resonance and ultrasound imaging to monitor the therapeutic process. Therefore, the nanosystem demonstrated a powerful multimodal combinational tumor therapy based on NO nanomedicine (Figure 9B) [105].

The ability of starvation therapy to induce hypoglycemia has also attracted the attention of researchers. Several studies have found that a low rate of NO in diabetic patients may be a contributing factors leading to systemic vascular lesions in diabetic nephropathy. Therefore, NO shows important application prospects as a therapeutic gas in the treatment of diabetic nephropathy. For example, Yang et al. designed an NO nanoprodrug by wrapping the NO donor L-Arg with magnetic nanoparticles as a shell and loading GOx on the surface. Under high glucose levels in blood, GOx on the surface of the nanosystem could react with glucose to reduce the level of glucose and generate H_2_O_2_. Under the action of the magnetic field, the NO donor could be oxidized by H_2_O_2_ to release NO gas, and then realize the therapeutic effect of NO gas. Finally, this strategy may be a potential therapeutic method for lowering blood glucose and alleviating the progression of diabetic nephropathy [180].

## 4. Conclusions and Outlook

As a burgeoning anti-tumor treatment method, gas therapy has attracted increasing attention—especially that of NO gas, owing to its unique disease treatment application in anti-tumor applications, which has been widely studied by researchers. To address the side effect of current common anti-tumor therapy, as well as the short half-life and poor stability of NO gas, it is of great significance to construct stimuli-responsive NO releasing nanomedicines. Based on the characteristics of NO donors, this paper summarizes the current progress of research into stimuli-responsive nanomedicines for the controlled release of NO, including via exogenous stimulation such as by light, ultrasound, X-ray and endogenous stimulation such as by pH, glucose and GSH. This paper also discussed the application of NO in the treatment of various diseases, especially in tumor treatment, and discussed the anti-tumor mechanism of NO-based nanodrugs in anti-tumor therapy and their combined therapy with chemotherapy, PDT, PTT, etc. However, the NO-based nanodrugs still have a long way to go before they can be clinically implemented.

Firstly, the continuous investigation of NO-based nanodrugs is a prerequisite for advancing the development of this field. Only by exploring stable and intelligent NO-based prodrugs can NO be released when stimulated, which ensured the on-demand release of NO at specifical locations and in specific required doses, avoiding the damage to normal tissues. However, it is difficult for NO donors to achieve complete stability without any leakage in the physiological environment while achieving complete release with the stimulation. Therefore, how to control the leakage dose of NO in a safe range becomes particularly important. This requires researchers to consider the tolerance of normal human cells to NO gas. Only in this way, the early release of a small amount of NO gas from NO-based nanomaterials may be acceptable as long as the upper limit is not exceeded. Secondly, it remains to ensure that the stimulus-responsive NO nanosystems can effectively target the desired sites, allowing NO to be released at a specific location without affecting normal tissues and cells. The biological barriers include rapid clearance in blood circulation, penetration into tumor sites, high interstitial pressure in tumor sites, tumor membrane barrier and lysosome/endosome barrier. These biological barriers impair the delivery efficiency of most nanomedicines, which limits their clinical transitions. Therefore, it is essential to design more effective delivery systems to improve the therapeutic efficiency. Although there have been various studies on the targeting of NO-based nanosystems, the versatility of these studies remains to be explored in the hope of finding a universal NO releasing strategy with a targeting effect on the vast majority of tumors. Third, actuating the evaluation of the biocompatibility and safety of the NO release system is the first prerequisite for the clinical application. For both endogenous and exogenous stimuli, we should ensure their effective controllability and safety to ensure the accurate release of NO. Therefore, there is also a need to develop multimodal imaging functions to achieve the accurate real-time monitoring of the position of the NO nanosystem and the accurate evaluation of the NO release. Given that immune therapy has shown its excellent anti-tumor effect and NO could increase the tumor immune response, investigators are reminded to further explore the therapeutic effect of NO therapy combined with immunotherapy. The combination of NO therapy with immunotherapy may prevent tumor recurrence and metastasis, which is also a very important research point. To sum up, a new tumor treatment strategy, NO gas therapy, needs us to constantly explore, which will bring hope for more patients, especially cancer patients, and a brighter prospects in the medical field.

## Figures and Tables

**Table 1:** Summary of the release mechanism and theranostic method of stimulus-responsive NO-based nanomedicine.

Trigger for NO Release	Nanomedicine Formulation	NO Donor	NO Release Mechanism	Theranostic Method	Reference
UV–Vis	mPEG–PLGA–BNN6–DOX	BNN6	The breaking of bonds	NO therapy–chemotherapy	[68]
	Cdot–TPP–SNO	R-SNO	The breaking of bonds	NO therapy	[69]
	Ru-NO@TiO_2_	Ru-NO	The breaking of bonds	NO therapy–PDT	[70]
NIR	PTNG	R-SNO	The breaking of bonds	NO therapy–PTT–chemotherapy	[71]
	PpRE@PEG–PpIX	PpRE	The breaking of bonds	NO therapy–PTT	[72]
	Fe_3_O_4_@PDA@Ru-NO@FA	Ru-NO	The breaking of bonds	NO therapy–PTT–MRI	[73]
	BNN–Bi_2_S_3_	BNN6	The breaking of bonds	NO therapy–mild PTT	[74]
	PNOC–PDA	R-SNO	The breaking of bonds	NO therapy–mild PTT–chemotherapy	[75]
	PEG–PAu@SiO_2_–SNO	R-SNO	The breaking of bonds	NO therapy–mild PTT	[76]
	PFTDPP–SNAP	SNAP	The breaking of bonds	NO therapy–PTT– NIR II/PA imaging	[77]
	Nb_2_C–MSNs–SNO	R-SNO	The breaking of bonds	NO therapy–PTT– PA imaging	[33]
	P(IR/BNN6/AIPH)@Lip–RGD	BNN6	The breaking of bonds	NO therapy–alkyl radicals–PTT	[78]
	RBCm/PAAV–SNO	R-SNO	The breaking of bonds	NO therapy–PTT–reprogramming tumor immunosuppressive microenvironment	[79]
	PDA–PLC/DOX	R-SNO	The breaking of bonds	NO therapy–PTT–chemotherapy	[80]
	Me–RBSs	RBS	The breaking of bonds	NO therapy	[81]
	GO–BNN6	BNN6	The breaking of bonds	NO therapy	[82]
	L-Arg@PCN@Mem	L-Arg	Oxidation–reduction reaction	NO therapy–PDT	[83]
	Arg–ZnPc	L-Arg	Oxidation–reduction reaction	NO therapy–PDT	[84]
	ADLAu@CuS YSNPs	L-Arg	Oxidation–reduction reaction	NO therapy–PDT–chemotherapy	[85]
	PLGA@ICG@L-Arg	L-Arg	Oxidation–reduction reaction	NO therapy–PDT	[86]
	Lyso–Ru-NO@FA@C–TiO_2_	Ru-NO	The breaking of bonds	NO therapy–PDT	[87]
US	BNN6–SPION@hMSN	BNN6	The breaking of bonds	NO therapy–MRI	[88]
	TPZ/HMTNPs–SNO	R-SNO	The breaking of bonds	NO therapy–SDT–USI	[89]
	IMesNO/DOX@MCs	IMesNO	The breaking of bonds	NO therapy–chemotherapy	[90]
	GSNO/Ce6@ZIF–8@Cytomembrane (GCZ@M)	GSNO	Oxidation–reduction reaction	NO therapy–SDT	[91]
	SNO–HSA–PTX	R-SNO	The breaking of bonds	NO therapy–chemotherapy–immunotherapy	[92]
	peptide−HMSN−LA	L-Arg	Oxidation–reduction reaction	NO therapy–SDT	[93]
X-ray	PEG–USMSs–SNO	R-SNO	The breaking of bonds	NO therapy–radiotherapy	[94]
	Bi–SNO	R-SNO	The breaking of bonds	NO therapy–radiotherapy–CT imaging–PTT	[95]
	ZGO:Mn–RBS	RBS	The breaking of bonds	NO therapy–radiotherapy	[96]
GSH	p(Gd–Az–JSK)	alkynyl-JSK	The nucleophilic attacking	NO therapy–chemotherapy	[97]
	PEG–b–NO-Dex–DOX	NO-Dex	Oxidation–reduction reaction	NO therapy–chemotherapy	[98]
	HCPT/CTS–NO–DMMA	PSF	Oxidation–reduction reaction	NO therapy	[99]
	α–CD–DOX–NO–DA	R-SNO	/	NO therapy–chemotherapy	[100]
	TNO3–DOX	TNO3	Oxidation–reduction reaction	NO therapy–chemotherapy	[101]
pH	hollow microsphere (HM)	DETA NONOate	Hydrolysis reaction	NO therapy–chemotherapy	[49]
	GSNO-MNPs	GSNO	Hydrolysis reaction–oxidation–reduction reaction	NO therapy–chemotherapy	[102]
	NO–NCPs	DETA NONOate	Hydrolysis reaction	NO therapy–PDT–PA imaging	[103]
Glucose	L-Arg–HMON–GOx	L-Arg	Oxidation–reduction reaction	NO therapy–starving therapy–USI	[104]
	BPNs–Arg–GOx@MnO_2_(BAGM)	L-Arg	Oxidation–reduction reaction	NO therapy–starving therapy–PTT	[105]

Abbreviations—BNN6: *N*,*N*′-di-sec-butyl-*N*,*N*′-dinitroso-1,4-phenylenediamine; R-SNO: S-nitrosothiol; Ru-NO: ruthenium nitrosyls; PpRE: porphyrin-modified Roussin’s red-salt ester; SNAP: S-nitroso-N-acetylpenicillamine; RBS: Roussin’s Black Salt([NH_4_][Fe_4_S_3_(NO)_7_]); L-Arg: L-arginine; IMesNO: 1,3-bis-(2,4,6-trimethylphenyl)imidazolylidene nitric oxide; GSNO: nitrosoglutathione; alkynyl-JSK: O_2_-(2,4-dinitrophenyl) 1-[4(propargyloxycarbonyl) piperazin-1-yl] diazen-1-ium-1,2-diolate; NO-Dex: hydrophobic nitrifying glucan; PSF: phenylsulfonyl furoxan; TNO_3_: nitrate functionalized D-α-tocopheryl polyethylene glycol succinate; DETA NONOate: diethylenetriamine diazeniumdiolate; PDT: photodynamic therapy; PTT: photothermal therapy; MRI: magnetic resonance imaging; NIR II/PA imaging: second NIR window and photoacoustic imaging; PA: photoacoustic; SDT: sonodynamic therapy; USI: ultrasound imaging; CT: computed tomography.
